# Single-cell transcriptomics reveals male germ cells and Sertoli cells developmental patterns in dairy goats

**DOI:** 10.3389/fcell.2022.944325

**Published:** 2022-07-22

**Authors:** Fa Ren, Huaming Xi, Pengyun Qiao, Yu Li, Ming Xian, Dawei Zhu, Jianhong Hu

**Affiliations:** ^1^ Department of Reproductive Medicine, Affiliated Hospital of Weifang Medical University, Weifang, China; ^2^ Key Laboratory of Animal Genetics, Breeding and Reproduction of Shaanxi Province, College of Animal Science and Technology, Northwest A&F University, Yangling, China

**Keywords:** single-cell RNA sequencing (scRNA-seq), dairy goat, spermatogenesis, Sertoli cell, testes

## Abstract

Spermatogenesis holds considerable promise for human-assisted reproduction and livestock breeding based on stem cells. It occurs in seminiferous tubules within the testis, which mainly comprise male germ cells and Sertoli cells. While the developmental progression of male germ cells and Sertoli cells has been widely reported in mice, much less is known in other large animal species, including dairy goats. In this study, we present the data of single cell RNA sequencing (scRNA-seq) for 25,373 cells from 45 (pre-puberty), 90 (puberty), and 180-day-old (post-puberty) dairy goat testes. We aimed to identify genes that are associated with key developmental events in male germ cells and Sertoli cells. We examined the development of spermatogenic cells and seminiferous tubules from 15, 30, 45, 60, 75, 90, 180, and 240-day-old buck goat testes. scRNA-seq clustering analysis of testicular cells from pre-puberty, puberty, and post-puberty goat testes revealed several cell types, including cell populations with characteristics of spermatogonia, early spermatocytes, spermatocytes, spermatids, Sertoli cells, Leydig cells, macrophages, and endothelial cells. We mapped the timeline for male germ cells development from spermatogonia to spermatids and identified gene signatures that define spermatogenic cell populations, such as AMH, SOHLH1, INHA, and ACTA2. Importantly, using immunofluorescence staining for different marker proteins (UCHL1, C-KIT, VASA, SOX9, AMH, and PCNA), we explored the proliferative activity and development of male germ cells and Sertoli cells. Moreover, we identified the expression patterns of potential key genes associated with the niche-related key pathways in male germ cells of dairy goats, including testosterone, retinoic acid, PDGF, FGF, and WNT pathways. In summary, our study systematically investigated the elaborate male germ cells and Sertoli cells developmental patterns in dairy goats that have so far remained largely unknown. This information represents a valuable resource for the establishment of goat male reproductive stem cells lines, induction of germ cell differentiation *in vitro*, and the exploration of sequential cell fate transition for spermatogenesis and testicular development at single-cell resolution.

## Introduction

Mammalian spermatogenesis involves the differentiation of spermatogonial stem cells into haploid spermatozoa, which is a complex and highly ordered process that plays a crucial role in the control of male fertility (De Oliveira et al., 2022). Cell division and gradual differentiation are the most basic biological events that occur during spermatogenesis. Information regarding how different cell types develop in this process is important for a clear understanding of spermatogenesis (Dunleavy et al., 2019). Postnatal mammalian spermatogenesis occurs in the seminiferous tubules, which are populated by male germ cells and Sertoli cells and canonically called niches (Savvulidi et al., 2019). Male germ cells undergo niche-guided transitions during diverse cellular processes and cell states, including mitosis, meiosis, and the stages of sperm deformation to maturation (Zhang et al., 2020). As the only somatic cell type in the niche that has direct contact with germ cells, Sertoli cells can produce a variety of growth factors that are necessary for the development of male germ cells during spermatogenesis (Zheng et al., 2022). Importantly, the advent of new sequencing technologies, such as single-cell RNA sequencing (scRNA-seq), has promoted the identification of the key genes controlling testicular cell development (Frost et al., 2021). Currently, the elucidation of gene expression dynamics during the development of male germ cells and Sertoli cells using single-cell transcriptomics has been reported in mice (Hermann et al., 2018; Tan and Wilkinson, 2020). However, molecules directing the male germ cells and Sertoli cells development by single-cell RNA sequencing in other large animal species including dairy goat, have not been identified.

Single-cell RNA sequencing can define the testicular transcriptomes at the single-cell level, while also describing the degree of cellular heterogeneity and the presence of rare subpopulations (Suzuki et al., 2019). Before scRNA-Seq, it was difficult to study on the systematic description of different developmental stages of male germ cells and somatic cells in farm animals at the single-cell level (Yang et al., 2021). Interestingly, scRNA-Seq technology can be used to explore cell heterogeneity and transcriptional changes that occur during spermatogenesis (Lau et al., 2020). Currently, single-cell transcriptomics of spermatogenesis has mainly focused on the testis cells of humans and mice (Wang et al., 2018; Cao et al., 2021). The molecular functions of spermatogonia, spermatocytes, spermatids, and testicular somatic cells as well as the heterogeneity among these cells have been reported (Suzuki et al., 2019). A recent study reported a single-cell transcriptome data of the testes of a pre-sexual young dairy goat, providing significant theoretical basis of spermatogenesis in buck goats (Yu et al., 2021). However, the development patterns of the male germ cells and Sertoli cells from pre-puberty to post-puberty have not been systematically investigated in dairy goats.

Dairy goat has been associated with humans for at least 10,000 years and considered a useful domestic animal (González-Marín et al., 2021). Owing to their environmental adaptability, these goats can increase the income of farmers. Moreover, the nutritional value of goat milk is high owing to its ease of digestion and absorption (Ren et al., 2021). This makes the dairy goat industry a major player in the dairy industry (Clark and Mora García, 2017). Additionally, dairy goats are also used as animal models for pre-clinical and translational studies because of their physiological and anatomical characteristics (Gul et al., 2020). To understand the development of male germ cells and Sertoli cells in dairy goats, we used the scRNA-Seq to comprehensively identify gene expression patterns in 25,373 individual cells from three dairy goat testes at different developmental stages. Furthermore, we performed histological and immunohistochemical analyses on testis sections at eight stages from pre-puberty to post-puberty. Taken together, we obtained a high-quality dataset, which provided an unbiased atlas of spermatogenesis-related cell populations at different stages of development in the dairy goat testis. The elucidation of the complex dynamics of developmental transitions provides valuable knowledge regarding the transcriptional landscape from spermatogonia to spermatids and offers a reference for dairy goat breeding.

## Materials and methods

### Chemicals and reagents

Unless otherwise stated, all chemicals and reagents used in this study were purchased from Sigma-Aldrich China.

### Animals used

**FIGURE 1:**
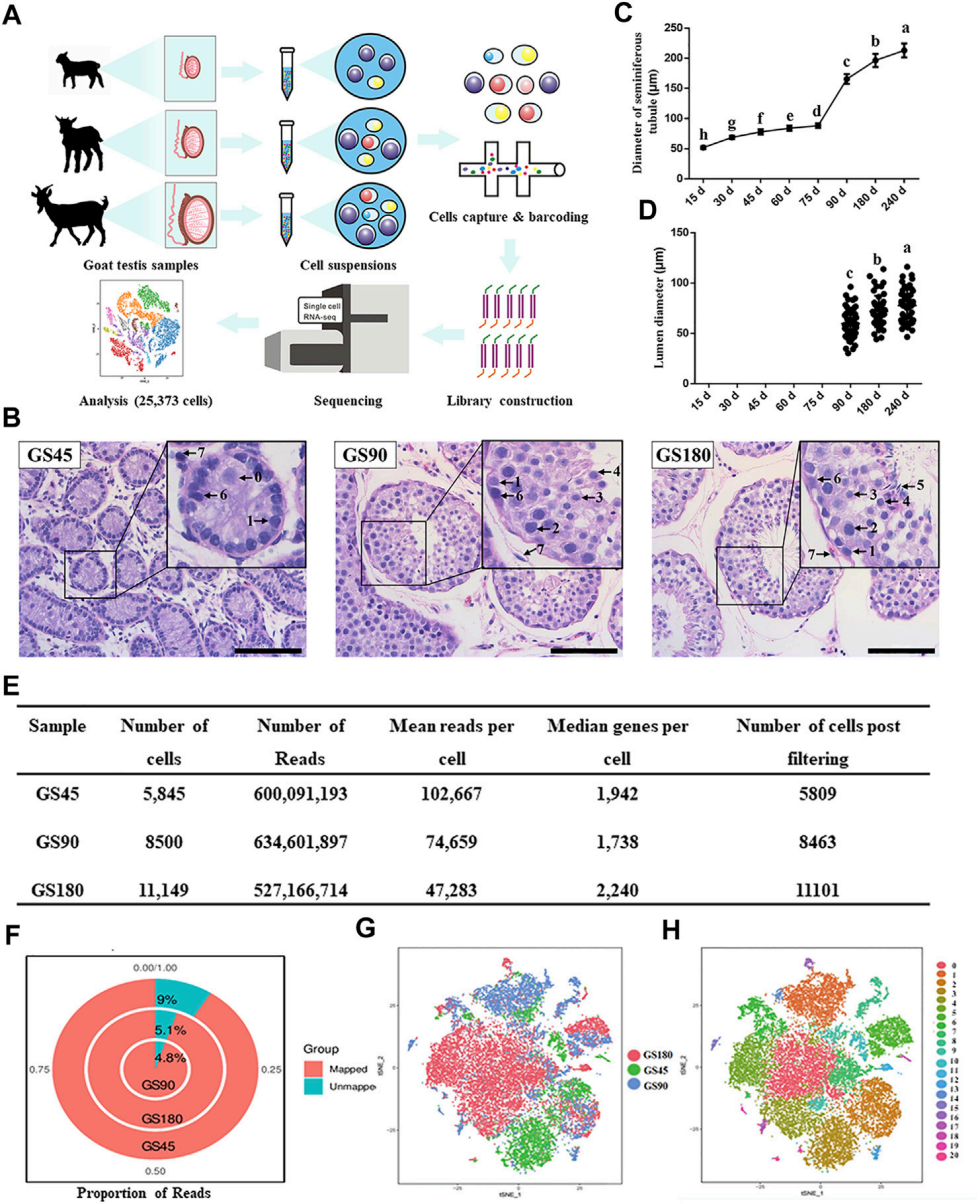
Overview of scRNA-seq in dairy goat testicular cells. **(A)** The single-cell RNA-seq process of dairy goat testicular cells. **(B)** Histomorphological analysis of dairy goat testes at different ages. 0, gonocyte. 1, spermatogonia. 2, early spermatocytes. 3, spermatocytes. 4, round spermatids. 5, elongated spermatids. 6, Sertoli cells. 7, Leydig cells. Bar = 100 μM. **(C)** Variation in the diameter of the seminiferous tubule of dairy goat testes. **(D)** Variation in the diameter of the lumen of the seminiferous tubule of dairy goat testes. **(E)** Single cell datasets quality metrics summary identified by Cell Ranger. **(F)** The reads mapped to genome of three samples. **(G)** Sample distribution result revealed in the tSNE plot. **(H)** Cell clustering revealed in the tSNE plot.

Twenty-seven Guanzhong dairy goat bucks with a similar genetic background and from the same breeding farm (Shaanxi Aonic Guanzhong Dairy Goat Breeding Co., Ltd., Fuping, China) born in February were used in this study. We collected testes at eight different ages from 24 Guanzhong dairy goats spanning from pre-puberty to post-puberty, that is, testes from 15, 30, 45, 60, 75, 90, 180, and 240-day-old goats (*n* = 3 each age). These testis samples were fixed in Bouin’s solution overnight at 4°C for histological and immunohistological analyses. Moreover, testis samples from Guanzhong dairy goats of three different ages were separated into single cells for scRNA-seq: 1) pre-puberty dairy goat testis (45-day-old, GS45), 2) puberty dairy goat testis (90-day-old, GS90), and 3) post-puberty dairy goat testis (180-day-old, GS180; Figure 1A). The surgical procedures were conducted by professional veterinarians, and all subsequent experiments were approved by the Institutional Animal Care and Use Committee of Weifang Medical University and Northwest A&F University.

### Histological analysis of seminiferous tubules

For histological analysis, paraffin-embedded testis tissues were cut into 5-μm sections, which were stained with hematoxylin-eosin (HE) as previously described (Xi et al., 2021). Briefly, testis sections were washed in phosphate buffered saline (PBS) twice after deparaffinization and rehydration, followed by HE staining using standard procedure, which included deparaffinization, permeabilization, antigen retrieval, blocking, primary antibody incubation, secondary antibody incubation, color development, and hematoxylin counterstaining. Next, the images of the sections were examined under a phase-contrast microscope (Nikon Eclipse C1). Based on the morphological characteristics of testicular cells, different cell types in the seminiferous tubules were identified as previously described (Xi et al., 2021). Next, the statistical analysis of the lumen diameter and diameter of seminiferous tubules at each age was carried out based on 50 round seminiferous tubule cross-sections from three replicated testicular samples (Figures 1C,D).

### Preparation of single cell suspensions

According to the method described by Ren et al. (2020), live castration of dairy goats was carried out to obtain complete testes. Single-cell suspensions were prepared as detailed below. Goat testes were collected and placed in ice cold PBS and transported to the laboratory on ice. After washing the testes thrice with PBS supplemented with 100 IU/ml penicillin and 100 μg/ml streptomycin, the visible connective tissue, tunica albuginea, and blood vessels were removed with forceps and a scalpel. After the removal of the epididymis, the testes were minced into small pieces and incubated with 1 mg/ml collagenase type IV (C5138, Sigma-Aldrich Corp, St. Louis, MO, United States) and 1 mg/ml hyaluronidase (H3056, Sigma-Aldrich Corp, St. Louis, MO, United States). Next, the mixture was mixed with Dulbecco’s modified eagle medium (DMEM, high glucose; Thermo Fisher Scientific) in equal volume, followed by incubation for 30 min at 37°C. The seminiferous tubules were collected through natural sedimentation, washed thrice with PBS, and then digested with trypsin (2.5 mg/ml) for 10 min at 37°C to isolate the cells. The enzymatic digestion process was carried out by adding DMEM containing 10% fetal bovine serum (Gibco). The cell suspension was filtered through a 40-μm mesh (BD Falcon, United States). After washing with PBS and centrifugation (thrice), the pellet was resuspended to obtain a single-cell suspension.

### Single-cell RNA-seq library preparation and sequencing

The single cell dairy goat Gel Beads-in-Emulsion (GEM) was prepared using the 10× Genomics Chromium platform according to the manufacturer’s instructions. Briefly, we adjusted the volume of the master mix according to the desired number of the captured cells and the concentration of the single-cell suspensions. Next, we placed the master mix, cell mixture, gel beads, and partitioning oil on the 10× controller chip for the reaction. As the Master Mix contained the enzymes needed for subsequent reverse transcription, we transferred the GEMs that were formed directly to polymerase chain reaction tubes for reverse transcription. After reverse transcription, the mRNA samples were purified using a Dynabeads™ MyOne™ Silane kit. cDNA synthesis, barcoding, and library preparation were then conducted using the protocol for the Chromium™ Single Cell 3ʹ Library and Gel Bead Kit v2. These libraries were sequenced on an Illumina NovaSeq 6000 PE150 System in a 2 × 150 bp paired-end mode.

### Analysis of primary single-cell data

We used FastQC to perform basic statistics on the quality of the raw reads. Next, the read sequences produced by the Illumina pipeline in FASTQ format were pre-processed using the Trimmomatic software, which consisted of the following steps: 1) Removing low-quality reads by scanning the reads with a four-base wide sliding window and cutting them when the average quality per base dropped below 10 (SLIDINGWINDOW: 4:10); 2) Removing trailing low quality or N bases (below quality 3; TRAILING:3); 3) Removing adapters using two modes, i.e., 1) alignment with the adapter sequence and removal when the number of matching bases was > 7 and mismatch = 2, and 2) removal of the non-overlapping portion when the overlapping base score for read1 and read2 was > 30 (ILLUMINACLIP: adapter. fa: 2: 30: 7); 4) Reads with ˂ 26 bases were dropped; 5) Reads that could not form pairs were discarded. The remaining reads that passed all the filtering steps were identified as clean reads and used in all subsequent analyses. Finally, we used FastQC to perform basic statistics regarding the quality of the clean reads.

### Alignment, barcode assignment, and UMI counting

We adopted Cell Ranger, which uses an aligner called STAR, to perform splicing-aware alignment of the reads to the genome. Cell Ranger used the transcript annotation GTF to bucket the reads into exonic, intronic, and intergenic and determined if the reads aligned to the genome. A read was considered exonic if at least 50% of it intersected an exon and intronic if it was non-exonic and intersected an intron; otherwise, it was considered intergenic. For reads that aligned to a single exonic locus but also aligned to one or more non-exonic loci, the exonic locus was prioritized and the read was considered confidently mapped to the exonic locus with MAPQ 255. Cell Ranger was further used to seek compatibility by aligning exonic reads to annotated transcripts. A read that was compatible with the exons of an annotated transcript and aligned to the same strand was considered mapped to the transcriptome. A read that was compatible with single gene annotation was considered uniquely mapped to the transcriptome. Only reads that were confidently mapped to the transcriptome were used for UMI counting.

### Dimensional reduction, clustering, and t-SNE projection

To reduce noise in the data and facilitate visualization, principal component analysis was used for dimensional reduction. After comparison and analyses, we found that for our data set, the ‘FindClusters’ function with resolution = 0.8 and dimension = 10, proved good clustering results. After clustering, the clustered cells were projected onto a two-dimensional (2D) map using the same principal components, and they were visualized by t-stochastic neighbor embedding (t-SNE). After t-SNE, the unique signature genes of each cluster were determined for subsequent cell type identification. In Seurat, the ‘FindAllMarkers’ function was built-in to calculate the signature genes of all the clusters. After the differentially expressed genes were identified, the downstream plotting function was used to visually analyze the differential genes. For example, the differentially expressed genes within each gene cluster were subjected to gene ontology (GO) analysis.

### Pseudo-time analysis

Pseudo-time analysis, also known as cell trajectory analysis, can be used to identify the differential trajectory of cells in the development process or the deformation of cell subtypes. The key genes in the cells were sorted in pseudo-time according to the expression pattern to simulate the dynamic changes of cells in the process of differentiation (Ge et al., 2021). Monocle 2 was used to perform the pseudo-time trajectories of qualified cells. In this study, based on the known differentiation relationship of male germ cell lineages, we selected nine kinds germ cell-related clusters and analyzed by Monocle 2. Briefly, according to the Monocle official website (https://cole-trapnell-lab.github.io/monocle-release/docs/#installing-monocle), all clusters have been reconstructed and analyzed, which used as ordering genes to order cells in pseudotime along a trajectory. Then, the plot_cell_trajectory function was used to visualize the differentiation trajectories of cells.

### Immunofluorescence

Testis samples were performed immunofluorescence as described (Ren et al., 2021). Briefly, the sections were deparaffinized, rehydrated, and infiltrated with 0.5% Triton X-100 for 10 min. The sections were then washed three times with PBS. Antigen retrieval was carried out in 1 × Tris/ethylenediamine tetraacetic acid (EDTA; pH = 9.0) for 20 min by boiling, followed by treatment with H2O2 (3%) for 10 min and washing (thrice) with PBS. Next, the sections were incubated with 10% donkey serum at 37°C for 2 h. They were then incubated with primary antibodies (Supplementary Table S1) or fluorescein isothiocyanate-peanut agglutinin (FITC-PNA, 100 μg/ml in PBS) in a humidified box overnight at 4°C. Next, the sections were washed thrice with PBS for 5 min per each section in a Rocker device, and the liquid was discarded. They were covered with secondary antibody and incubated for 50 min in the dark at 37°C. The sections were then immersed in EDTA antigen retrieval buffer to remove the primary and secondary antibodies. Slides were incubated with secondary antibodies overnight at 4°C. Objective tissue was covered with secondary antibody (Supplementary Table S1) and incubated in the dark for 50 min at 37°C. Next, they were incubated with 4, 6-diamidino-2-phenylindole solution at 37°C for 10 min. Section staining was monitored and photographed using an epifluorescence microscope (Nikon 80i; Nikon, Tokyo, Japan). Next, the statistical analysis of positive cells per cross-section of seminiferous tubules at each age was conducted based on 9 round seminiferous tubule cross-sections from three replicated testicular samples, respectively.

### Statistical analyses

All quantitative results are expressed as the mean ± standard error of the mean. Statistical analyses of data were carried out using one-way ANOVA, and multiple comparisons were performed with Duncan’s multiple range tests using SPSS version 22.0 for Windows (SPSS Inc., Chicago, IL, United States). Different letters in the same group of data indicate statistical significance at *p* < 0.05.

## Results

Overview of scRNA-seq and histological analysis of spermatogenic cells in postnatal goat testes.

To investigate the development of spermatogenic cells in dairy goat, we conducted histological analysis to examine morphological changes in spermatogenic cells during postnatal development. Testes were collected from 15, 30, 45, 60, 75, 90, 180, and 240-day-old dairy goats and testicular sections were stained with HE. We observed that the diameter of the seminiferous tubules increased significantly as the buck goats grew, and the lumens were observed for the first time in the samples collected from the 90-day-old goats (Figures 1B–D, Supplementary Figure S1A). In the 45-day-old testes samples, male germ cells were observed in the center of seminiferous cords, and we found that the morphology of the cells was similar to that of gonocytes (Figure 1B). In the 90-day-old samples, round spermatids appeared, indicating that buck goats had entered peri-puberty (Figure 1B). ​Moreover, all the types of spermatogenic cells appeared at 180 days after birth, which included spermatogonia, spermatocytes, as well as round and elongated spermatids (Figure 1B).

Next, we used the 10x Genomics Chromium platform to construct single cell RNA-seq libraries using 25,494 testicular cells from GS45, GS90, and GS180 (Figure 1A). We used physical methods to remove a large number of testicular somatic cells, followed by scRNA-seq and integrated data. To eliminate potentially low-quality data caused by technical issues, such as broken cells and multiplets, we set thresholds for data for the three testicular cells using quality metrics, and retained high-quality data of 25,373 cells (Figure 1E). The quality control analysis of the data from the three samples were in proportion to mitochondrial UMIs; the total number of UMIs detected in a single cell were similar among the three samples, indicating small technical differences among the samples (Supplementary Figure S1B). Additionally, all three testicular samples had high mapping rates, and were mapped to the goat genome at 91% (GS45), 95.2% (GS90), and 94.9% (GS180) (Figure 1F). We successfully batch-corrected the cell data of the three samples, which were almost blended when projected in the t-SNE space (Figure 1G). To group cells with similar gene expression, the data were analyzed using Seurat’s unsupervised clustering method to cluster cells into subpopulations based on the FindClusters function (resolution = 0.8, dimension = 10) and detected 21 clusters (Figure 1H). In summary, we generated high-quality scRNA-Seq data in dairy goat testicular cells from the pre-puberty, peri-puberty, and post-puperty.

### Identification and cluster analysis dairy goat testes cell types

**FIGURE 2:**
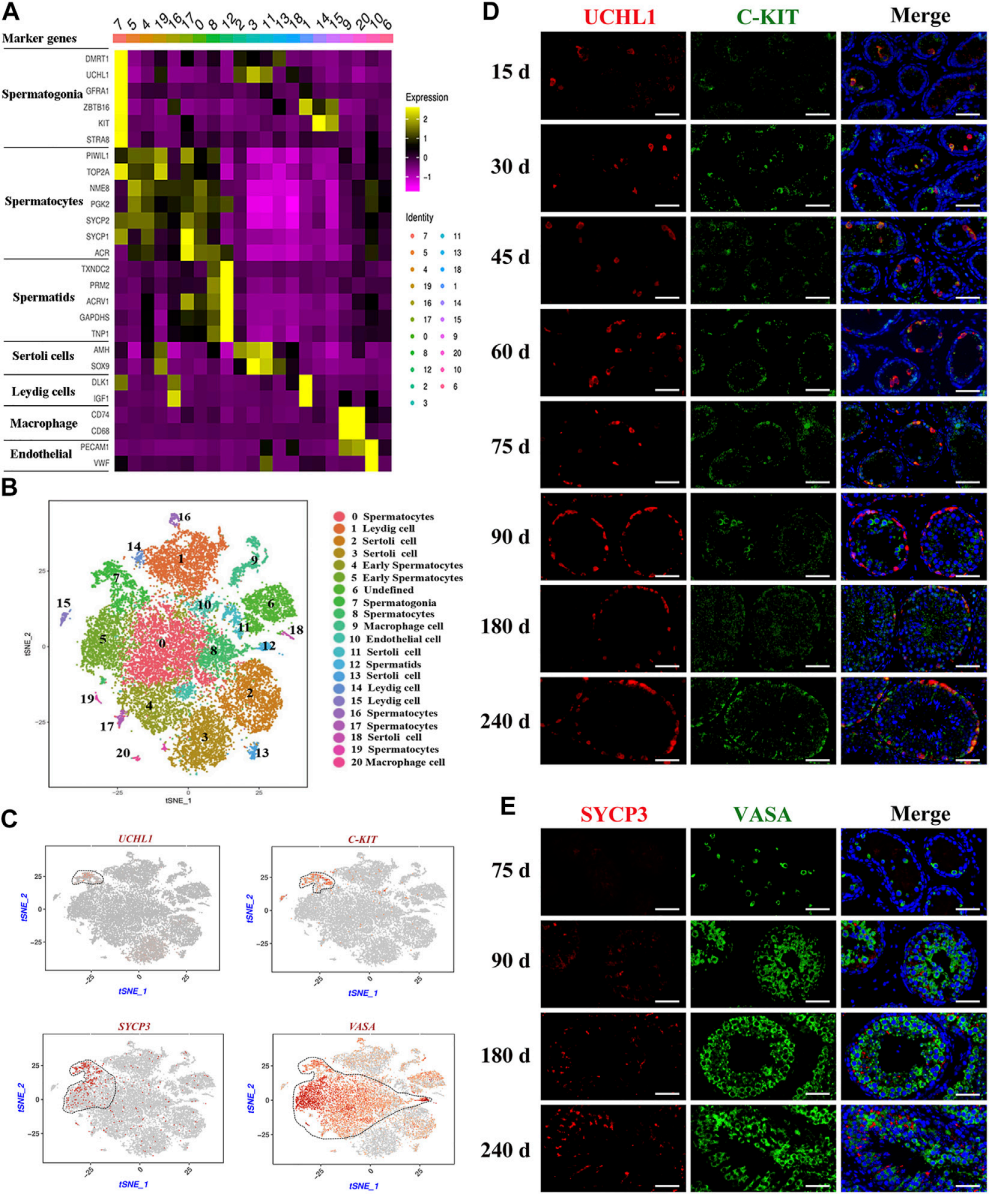
Identification of cell types during spermatogenesis and male germ cells development in dairy goat testes. **(A)** The marker genes specifically expressed of the cell cluster in heat map. **(B)** Cell clusters identification in the tSNE plot. **(C)** Expression patterns marker genes for male germ cells visualized in t-SNE plots. **(D)** UCHL1 and C-KIT immunostaining of dairy goat testis sections at each age. Bar = 100 μM. **(E)** SYCP3 and VASA immunostaining of dairy goat testis sections at 75 to 240-day-old. Bar = 100 μM.

To further identify the goat testicular cell types in each cluster, we selected the marker genes based on known cell-type markers. Spermatogonia specific markers DMRT1, GFRA1, ZBTB16, and STRA8 were highly expressed in cluster 7 (Figure 2A, Supplementary Figure S2A). TOP2A, SYCP1, SYCP2, and NME8 were expressed in clusters 0, 4, 5, 8, 16, 17, and 19. Subsequently, these seven cell clusters were identified as two stages of spermatocytes along the spermatogenesis transition (clusters 4 and 5: early spermatocytes; clusters 2, 8, 16, 17, and 19: spermatocytes) (Figure 2A, Supplementary Figure S2A). Spermatid specific markers were expressed solely in cluster 12 (e.g., TNP1, GAPDHS, PRM2, and ACRV1) (Figure 2A, Supplementary Figure S2A). Among the testicular somatic cells (niche), clusters 2, 3, 11, 13, and 18 were Sertoli cells, which expressed SOX9 and AMH. Clusters 1, 14, and 15 were testicular Leydig cells, with the exclusive expression of IGF1 and PDGFRA. Clusters 9 and 20 included macrophages, for which CD74 and CD68 were expressed. Cluster 10 corresponded to endothelial cells, with the expression of PECAM1 and VWF (Figure 2A, Supplementary Figure S2B). According to the expression of key cell-type-specific markers for goat testicular cells, we identified 21 clusters of cells in goat testis, including nine male germ cell clusters, and the type for cluster 6 was unknown (Figure 2B).

### Development of male germ cells in dairy goat testes

To further confirm the results of scRNA-seq cluster analysis and explore the development that occurs from spermatogonia to spermatid differentiation during the early life of dairy goats, we analyzed the marked germ cells using immunohistology. We found that the VASA-positive clusters in the scRNA-seq dataset contained all types of goat male germ cell clusters (Figure 2C). Among the VASA-positive clusters, cluster 7 comprised spermatogonia, which specifically expressed UCHL1 and C-KIT (KIT). Cluster 5 comprised early spermatocytes, with the exclusive expression of SYCP3 (Figure 2C). Immunostaining was performed to validate the expression patterns of UCHL1, C-KIT, SYCP3, and VASA. In the 15-day-old samples, UCHL1-positive and C-KIT-positive cells were only observed in the center of seminiferous tubules and not attached to the basal membranes (Figure 2D). All UCHL1-positive cells were distributed on the basement membrane in the 75-day-old samples, indicating that gonocytes completed migration and became spermatogonia. Additionally, UCHL1-negative and C-KIT-positive cells were observed in the 30–240-day-old samples, suggesting that spermatogonia began to differentiate at 30 days (Figure 2D). Next, we found that VASA-positive cells were negative for SYCP3 in the 75-day-old samples (Figure 2E). However, in the 90-day-old samples, SYCP3-positive cells began to emerge, indicating that the development of meiotic germ cells occurred from 75 to 90 days after birth (Figure 2E). The results of co-staining with VASA and PNA (a sperm-acrosome-specific marker) showed that PNA-positive cells and VASA-positive cells were present in the 90-240-day-old samples. Interestingly, all round spermatids were VASA-positive cells, while elongated spermatids were VASA-negative cells, suggesting that VASA is not a marker for elongated spermatids (Supplementary Figure S2C).

### Proliferative activity of spermatogonia in dairy goat testes

**FIGURE 3:**
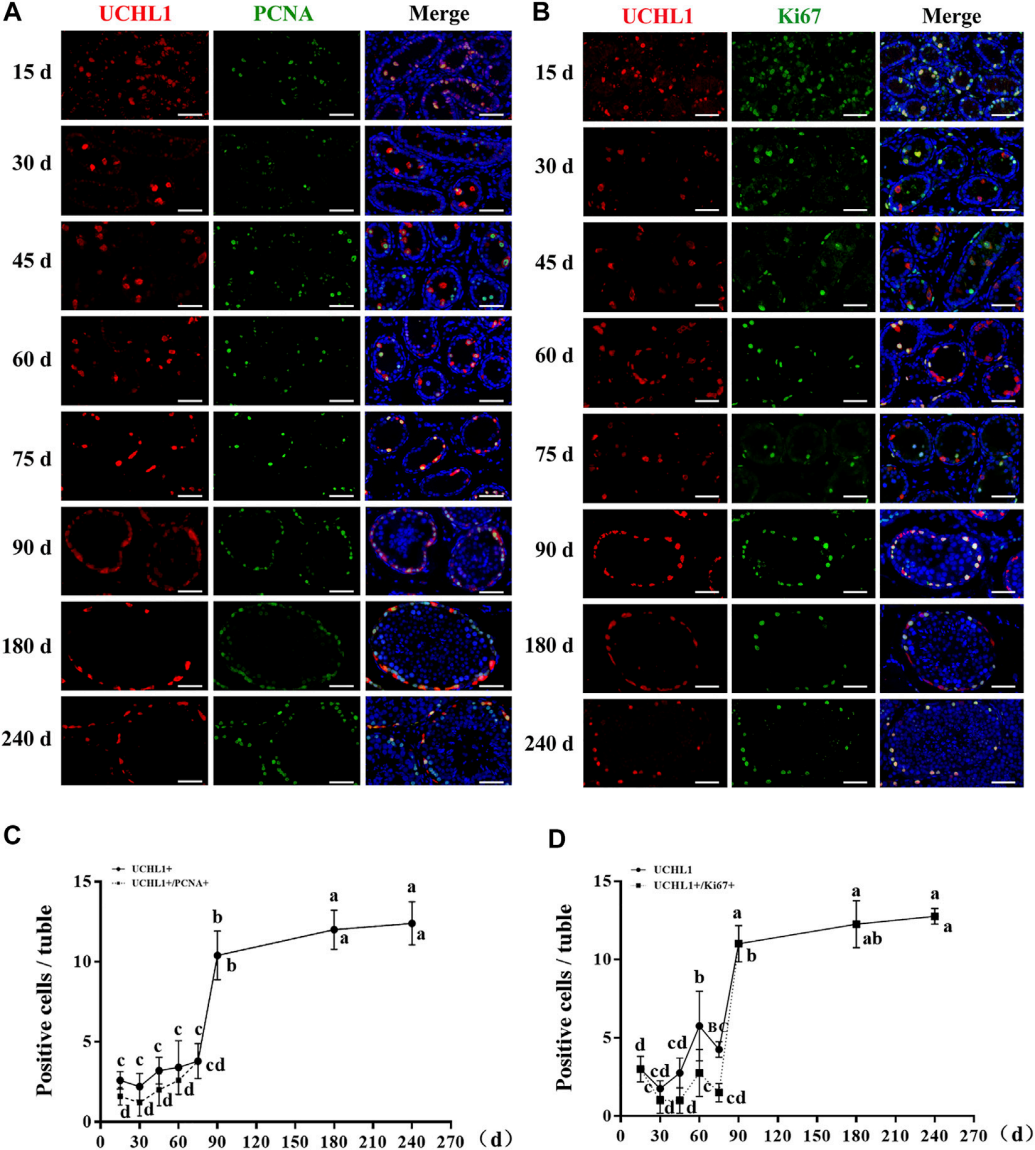
The proliferative activity of spermatogonia in dairy goat testes. **(A)** UCHL1 and PCNA immunostaining of dairy goat testis sections at each age. Bar = 100 μM. **(B)** UCHL1 and Ki67 immunostaining of dairy goat testis sections at each age. Bar = 100 μM. **(C)** The numbers of UCHL1/PCNA-positive cells per cross-section of seminiferous tubules in dairy goat testes at each age (*n* = 9). **(D)** The numbers of UCHL1/Ki67-positive cells per cross-section of seminiferous tubules in dairy goat testes at each age (*n* = 9). Different letters imply significant difference (*p* < 0.05), and same letters mean no significant difference (*p* > 0.05).

Subsequently, to probe the proliferative activity of spermatogonia in dairy goat testes, we performed double immunostaining for UCHL1 and PCNA/Ki67 on testis sections. Immunofluorescence staining results showed that both UCHL1+PCNA+ and UCHL1+Ki67 + cells were present at each age (Figures 3A,B). However, UCHL1-positive and PCNA-negative cells were present in 15–75-day-old dairy goat testes (Figure 3A). Notably, in the 90–240 days old samples, all UCHL1-positive cells expressed PCNA protein, suggesting that spermatogonia were beginning to enter the active stage of proliferation, and this was consistent with the results of UCHL1 and Ki67 co-staining (Figures 3A,B). Quantification of cells positive for UCHL1 and PCNA per cross-section revealed that the number of UCHL1-positive and PCNA-positive cells increased rapidly, and the slope was the largest in 75–90-day-old testes samples (Figure 3C). Similarly, the numbers of UCHL1-positive and Ki67-positive cells consistently grew with age, peaking at 90 days after birth (Figure 3D).

### Cell marker gene identification in goat testicular cell types

**FIGURE 4:**
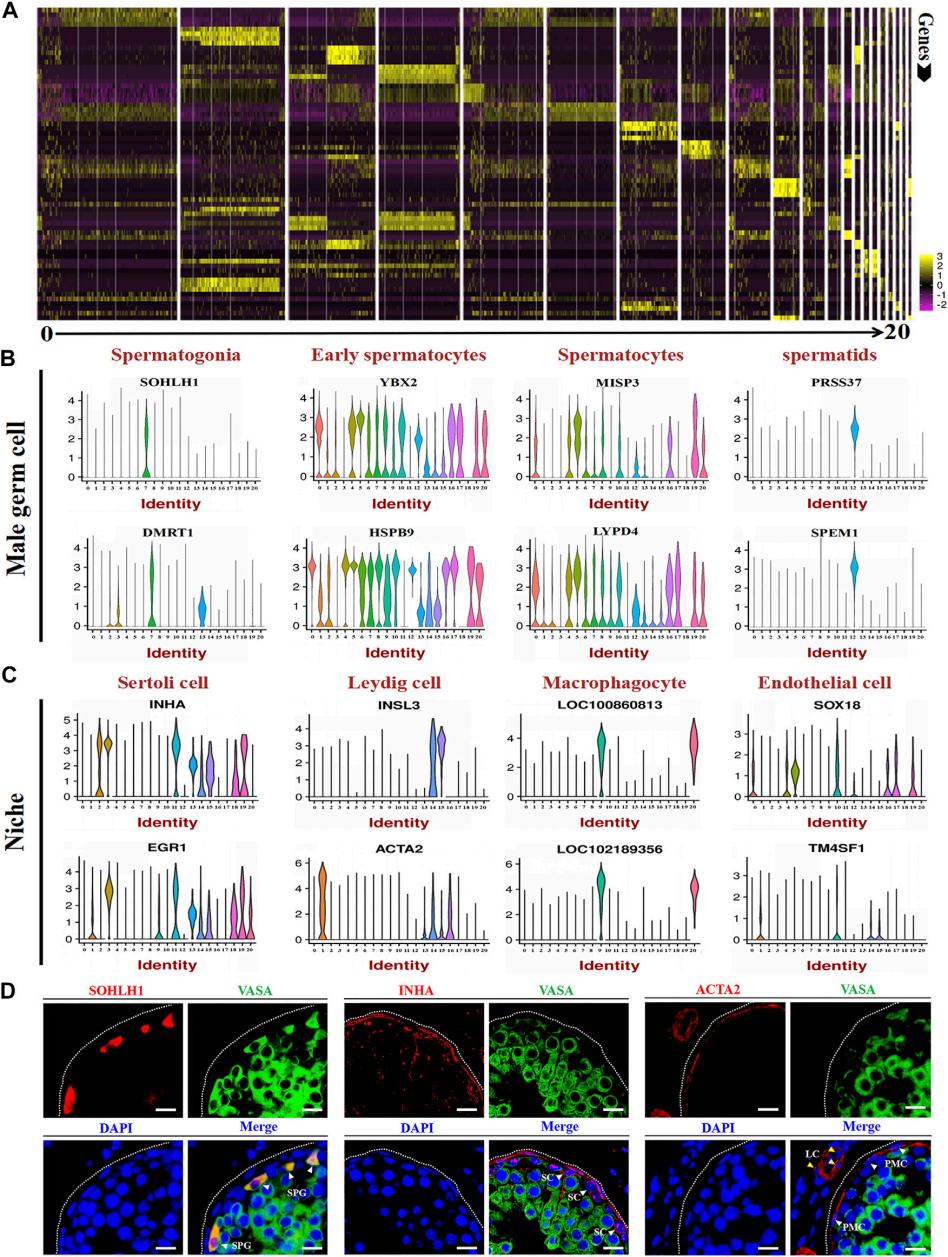
Marker gene identification of each testicular cell type. **(A)** Heatmaps show the top 4 significantly differentially expressed genes between each cell cluster for testicular cells. **(B,C)** Expression patterns (violin plot) of each cell specific gene across the different cluster. **(D)** Immunofluorescence analysis the expression location of SOHLH1, INHA, and ACTA2 in dairy goat testes. Three potential markers (SOHLH1, spermatogonia; INHA, Sertoli cells; ACTA2, testicular somatic cells) and male germ cell marker (VASA) were stained in adult testis tissue sections, respectively. The DAPI (blue) denotes nuclei. SPG, spermatogonia. SC, Sertoli cell. LC, Leydig cell. PMC, peritubular myoid cells. Bar = 10 μm.

After identifying different clusters of cell types in the single-cell dataset, we determined the gene expression profile of each cluster. As shown in Figure 4A, a heat map illustrated the expression of the top 4 marker genes; detailed information for these genes is listed in Supplementary Table S2. To identify the expression of the marker genes in male germ cells and their niche, we randomly selected two marker genes for each cell type for gene expression analysis per cluster. The results showed that SOHLH1, DMRT1, YBX2, HSPB9, MISP3, LYPD4, PRSS37, and SPEM1 were highly expressed in spermatogonia, early spermatocytes, spermatocytes, and spermatids (Figure 4B). INHA, EGR1, INSL3, ACTA2, LOC100860813, LOC102189356, SOX18, and TM4SF1 were highly expressed in different niches (Figure 4C). The immunofluorescence staining experiment also verified the expression location of SOHLH1, INHA, and ACTA2 in adult goat testes (Figure 4D). Importantly, all SOHLH1-positive cells were distributed on the basement membrane and completely coincided with VASA-positive cells, suggesting SOHLH1 could be used as a potential spermatogonia marker for dairy goat (Figure 4D).

Cell trajectory analysis and patterns of coordinated gene expression of male germ cell in dairy goat testes.

**FIGURE 5:**
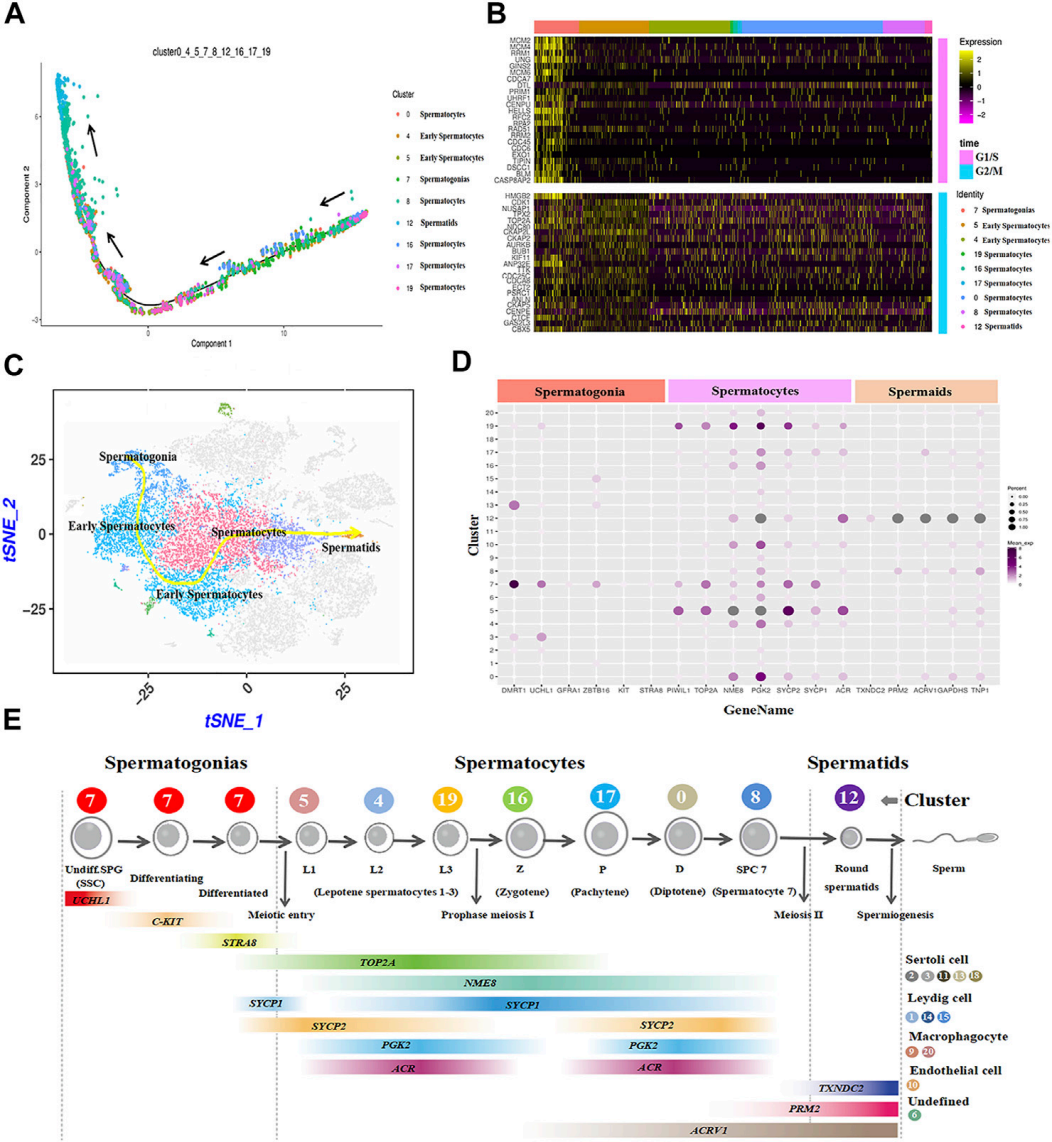
Construction of germ cell differentiation lineage in dairy goats and regulating gene expression patterns. **(A)** Cell trajectory analysis of nine testicular germ cell types. **(B)** Heat map of cell-cycle-specific genes in male dairy goat germ cells. **(C)** The indicator map of germ cell developmental changes. **(D)** Dot plot illustrating of germ cells key genes expressed in cell clusters. **(E)** Identification of cell clusters expressing the noted marker genes allowed clusters to be aligned with specific spermatogenic cell types.

The cell trajectory analysis of male goat germ cells was determined by Monocle. Nine cell types for spermatogenesis were clustered and identified, and the unsupervised pseudo-time exhibited the developmental trajectory of male goat germ cells, which occurred from spermatogonia to spermatids (Figure 5A). Then, the germ cell cycle status was assessed by analyzing the G1/S and G2/M phase-specific genes, as shown in Figure 5B. The cell proliferation status of G1/S and G2/M phase-specific genes was active in spermatogonia. Furthermore, reduced expression of G1/S phase-specific genes was detection in spermatocytes. G2/M phase-specific genes were relatively more active in spermatocytes than G1/S-phase-specific genes (Figure 5B). After meiosis Ⅱ, spermatids exited the cell cycle, entered sperm deformation stage, and were transferred to the epididymis (Figure 5B). We also mapped the developmental path of nine germ cell clusters, ranging from spermatogonia to sperm (Figure 5C). To further explore the patterns of coordinated gene expression and changes during goat spermatogenesis, we used dotplot to compare and analyze the marker genes of each germ cell cluster (Figure 5D). Based on the results and the spermatogenesis process, cluster 7 was identified as spermatogonia; clusters 5, 4, and 19 were leptotene spermatocytes 1-3; cluster 16 was zygotene (Z); cluster 17 was pachytene (*p*); cluster 0 was diplotene (D); cluster 8 was spermatocyte 7 (SPC7), and cluster 12 was spermatids (Figure 5E). Fixing the order of male germ cell clusters along pseudo-time, the marker genes of each germ cell cluster were annotated with GO (Supplementary Figure S3) and Kyoto encyclopedia of genes and genomes (KEGG) (Supplementary Figure S4) databases to analyze the differences between each cell type.

### Sertoli cell development in dairy goat testes

**FIGURE 6:**
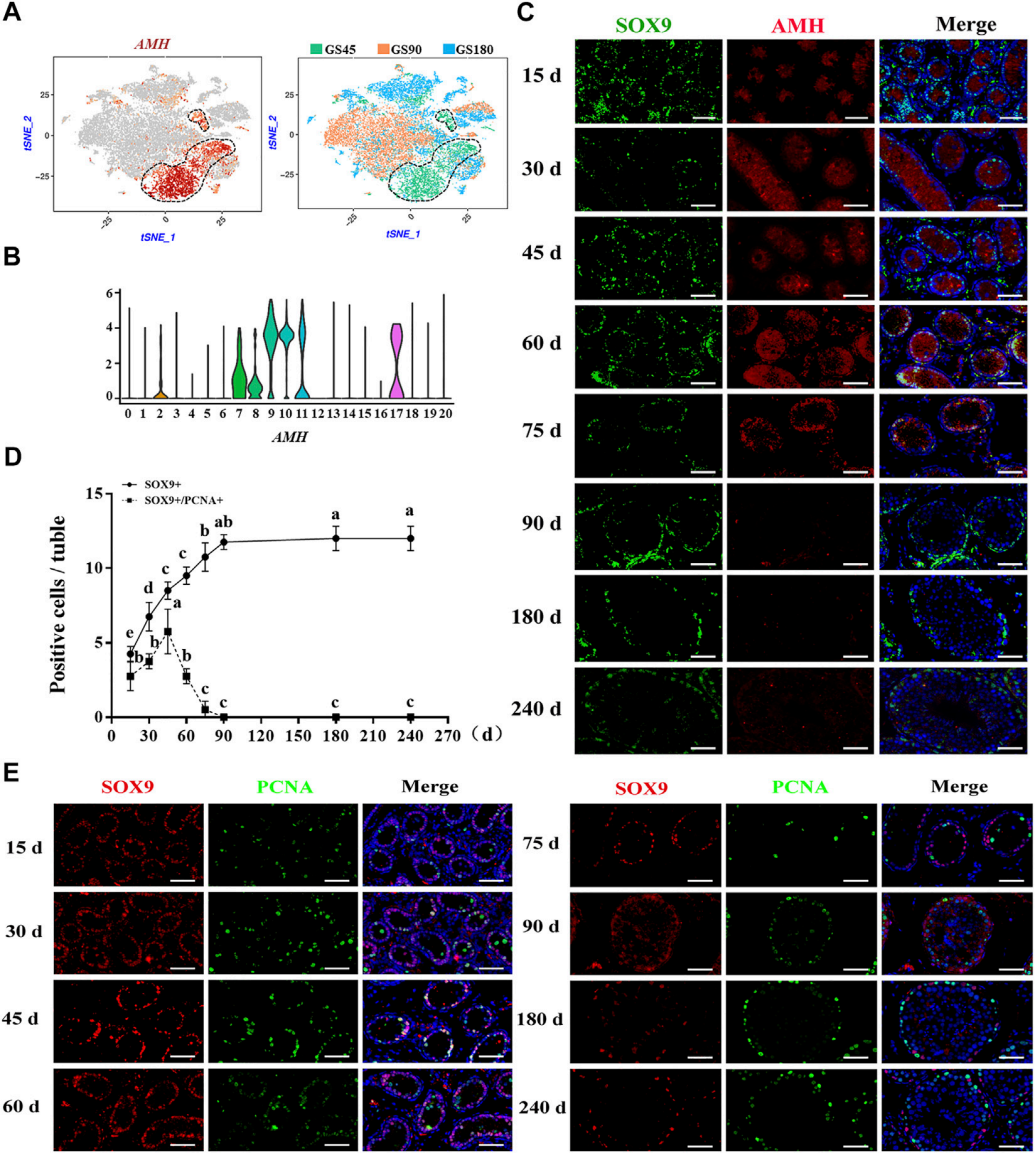
Sertoli cells development in dairy goat testes. **(A)** Expression patterns AMH for Sertoli cells visualized in t-SNE plots. **(B)** Expression patterns (violin plot) of AMH the different cluster. **(C)** AMH and SOX9 immunostaining of dairy goat testis sections at each age. Bar = 100 μM. **(D)** The numbers of SOX9/PCNA-positive cells per cross-section of seminiferous tubules in dairy goat testes at each age (*n* = 9). Different letters imply significant difference (*p* < 0.05), and same letters mean no significant difference (*p* > 0.05). **(E)** SOX9 and PCNA immunostaining of dairy goat testis sections at each age. Bar = 100 μM.

Subsequently, the development of Sertoli cells was explored in dairy goat testes. Interestingly, we found that AMH was only expressed in the clusters of GS45, suggesting AMH was only expressed in immature Sertoli cells of dairy goat testes (Figures 6A,B). Next, to identify and probe the development of Sertoli cells, we incubated the cells with SOX9 and AMH antibodies. Co-immunofluorescence staining results showed that AMH was present in the testes of dairy goats that were 15–75 days old, but absent in the 90-day-old goats, indicating that dairy goat Sertoli cells become mature before the goats turned 90 days old (Figure 6C). However, SOX9-positive cells were observed in dairy goat seminiferous tubules at all ages (Figure 6C). To pinpoint the proliferative activity of Sertoli cells in dairy goat, we performed double immunostaining for SOX9 and PCNA on testis sections. Immunofluorescence staining results showed that both SOX9 and PCNA-positive cells were present in 15 to 75-day-old dairy goat testes samples, but that only SOX9-positive cells could be observed in 90-day-old testes, indicating that Sertoli cells cease to proliferate in peri-puberty (Figure 6E). In addition, we found that the number of SOX9-positive cells substantially increased between 15 and 75 days after birth and that they would possibly not proliferate in the testes between 90 and 240 days (Figure 6D).

### Male germ cells and niche-related gene expression of key pathways in dairy goat

**FIGURE 7:**
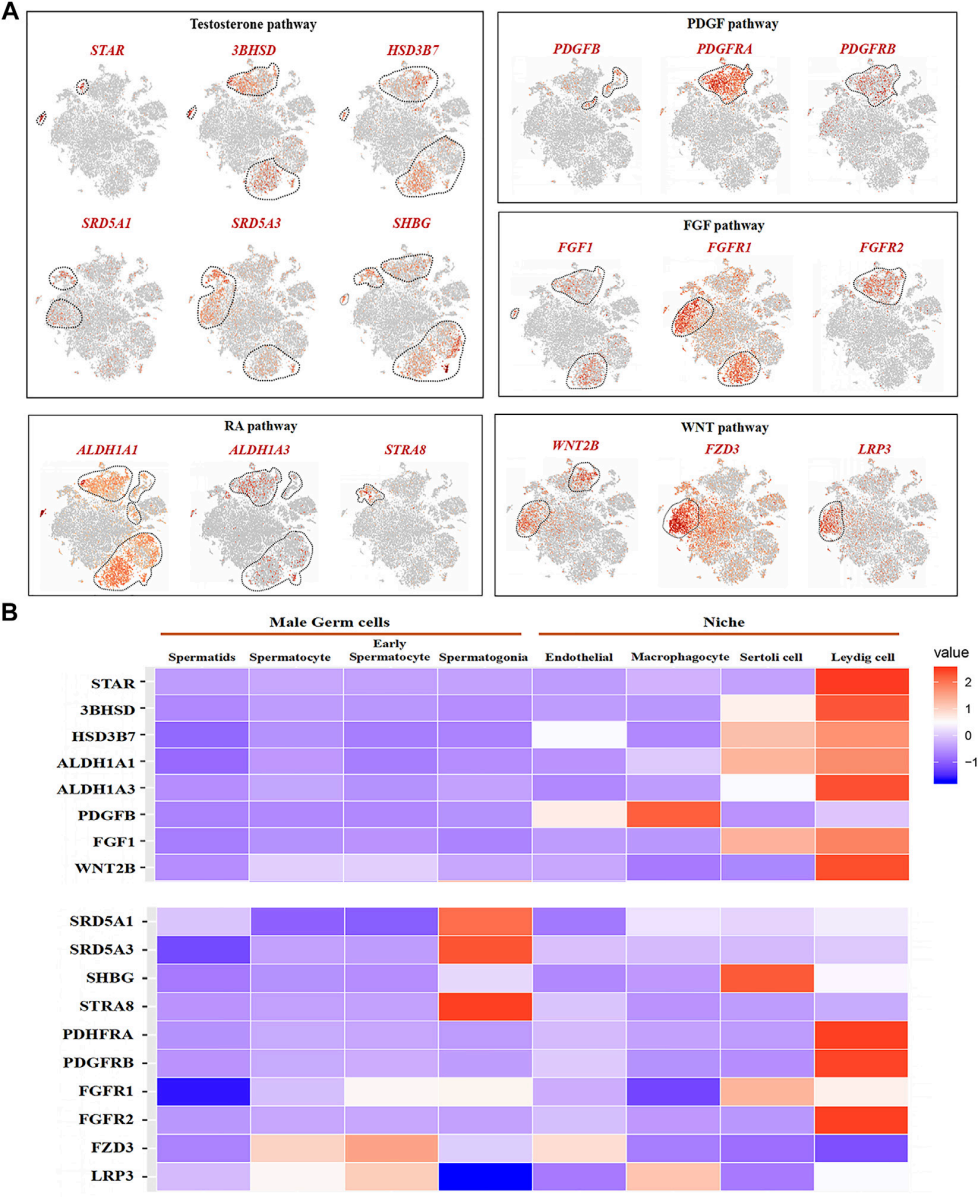
Male germ cells and niche-related gene expression of key pathways in dairy goat. **(A)** Male germ cells and niche-related gene expression in the tSNE plot. **(B)** Heat map illustrating showed related genes in the male germ cells and Niche respectively.

**FIGURE 8:**
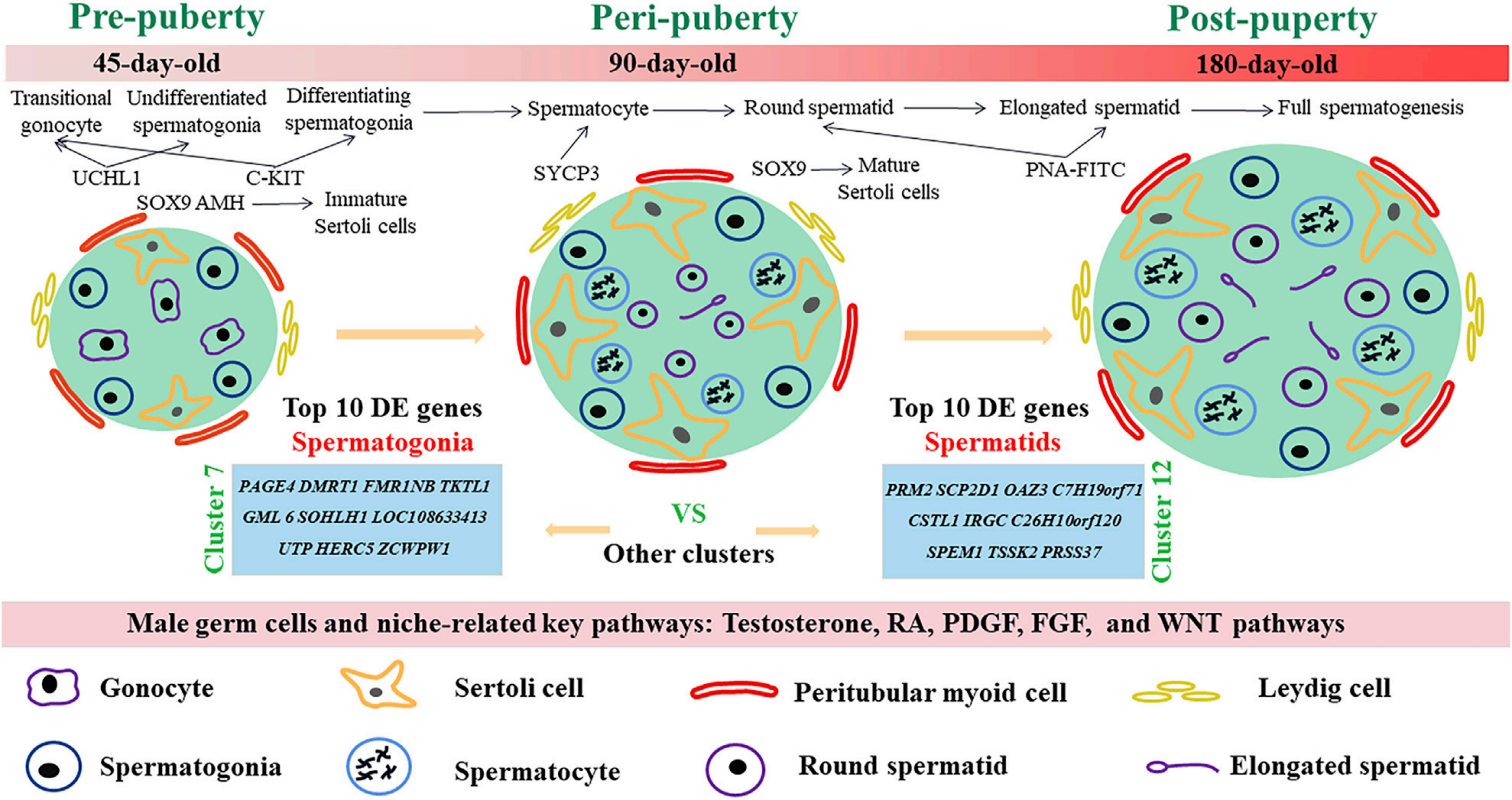
Schematic diagram of postnatal male germ cells and Sertoli cells development dynamics in dairy goat.

We analyzed the related gene expression of key pathways and determined that the key genes for testosterone biosynthesis, STAR, 3BHSD, and HSD3B7, were expressed in Leydig and Sertoli cells, and their response genes, SRD5A1, SRD5A3, and SHBG, were expressed in spermatogonia, early spermatocytes, and Sertoli cells (Figure 8A). ALDH1A1 and ALDH1A3, encoding enzymes retinoic acid (RA) synthesis, were specifically expressed in Sertoli and Leydig cells. The RA target gene, STRA8, was only observed in spermatogonia (Figure 7A). Interestingly, PDGFB was expressed in macrophage and endothelial cells, and its receptors, PDGFRA and PDGFRB, were found in Leydig cells (Figure 7A). FGF1 was expressed in Sertoli and Leydig cells, and its receptors, FGFR1 and FGFR2, were in early spermatocytes, Sertoli, and Leydig cells (Figure 7A). In the WNT pathway, the ligand WNT2B was primarily expressed in early spermatocytes and Leydig cells, and the receptors, FZD3 and LRP3, were primarily expressed in early spermatocytes (Figure 7A). Further analysis of these key genes, revealed that most of the ligand genes related to testicular somatic cell function during spermatogenesis were highly expressed in Sertoli and Leydig cells. Additionally, their receptor genes were highly expressed in spermatogonia and early spermatocytes (Figure 7B).

## Discussion

In this study, we examined the developmental patterns of male germ cells and Sertoli cells during the early life of dairy goat by using scRNA-seq and immunohistochemical analyses. We charted the elaborate developmental patterns of male germ cells and Sertoli cells in dairy goat from pre-puberty to post-puberty (Figure 8). We also defined nine different germ cell clusters and four kinds of somatic cells, screened several marker genes, and elucidated the complex relationships of potential key genes in male germ cells and testicular somatic cells during spermatogenesis in dairy goats. Together, our study provides a useful resource for the understanding of spermatogenesis and postnatal testicular development in dairy goat.

Previous studies have demonstrated that a lot of qualitative and quantitative changes take place in mammalian testis during postnatal development (Sararols et al., 2021). A recent report on testicular development in pigs stated that the diameter of seminiferous tubules size grows with age and that Duroc pigs reach puberty at 150 days old (Zheng et al., 2022). In Alpine goat, the seminiferous tubules lumen, spermatocytes, and spermatozoa emerge at 4 months (Faucette et al., 2014). However, our results showed that spermatocytes, round spermatids, and the seminiferous tubules lumen were identified in 90-day-old dairy goat testes, suggesting that Guanzhong dairy goat can reach puberty at approximately 90 days after birth. Notably, goat testicular development could vary with breed, individual, as well as feeding condition and management level, which might lead to differences in the key time nodes of seminiferous tubule development.

In this study, 25,373 cells were divided into 21 clusters through t-SNE (Linderman et al., 2019). Among these, nine clusters were identified as germ cells. The cluster 7 was identified as spermatogonia, specifically expressing DMRT1 and GFRA1. In a previous study, DMRT1 and GFRA1 were spermatogonia stem cells marker genes in dairy goat, respectively (Wei et al., 2021; Song et al., 2015). Moreover, STRA8 was considered to be a differentiation spermatogonia marker gene for mice and played an important role in meiosis during goat spermatogenesis (Yao et al., 2014). Thus, cluster 7 contained SSCs, differentiating spermatogonia, and differentiated spermatogonia, which is similar to the results obtained by previous report (Wang et al., 2018). There are few reports on goat spermatocyte and spermatid marker genes, which posed difficulties when defining germ cell clusters. Based on the spermatocyte marker genes (e.g., NME8, SYCP1, SYCP3) of humans and mice, clusters 5, 4, and 19 were identified as leptotene spermatocytes 1-3, cluster 16 was zygotene, cluster 17 was pachytene, cluster 0 was pachytene, cluster 8 was SPC7 (contained diakinesis, metaphase, anaphase, telophase, and secondary spermatocytes), and cluster 12 was spermatids (Wang et al., 2018). These results are consistent with the current reports on rodents and primates, and illustrate the similarity among male germ cell types in mammalian spermatogenesis.

To study the onset of male germ cell development in dairy goats, we performed co-staining for UCHL1/C-KIT, UCHL1/PCNA, UCHL1/Ki67, SYCP3/VASA, and PNA/VASA on goat buck testis sections. UCHL1 and C-KIT are undifferentiated and differentiated spermatogonia marker protein in goats, respectively (Heidari et al., 2012). Interestingly, we found that UCHL1-positive and C-KIT-positive cells were only observed in the center of seminiferous tubules, suggesting that UCHL1 and C-KIT could be used as marker proteins of gonocytes in dairy goat, which is similar to previous reports (Lee et al., 2018). In addition, the C-KIT mRNA and protein profile have been reported expression in normal rat testes gonocytes, which is consistent with our reports (Prabhu et al., 2012). Meanwhile, UCHL1-negative and C-KIT-positive cells were observed in 30-day-old samples, suggesting that spermatogonia began to differentiate at this age. Spermatogenesis depends on the proliferation and differentiation balance of undifferentiated spermatogonia. PCNA and Ki67 are expressed in the nucleus of cells as two prevalent cell proliferation markers (Zheng et al., 2022). We identified that UCHL1-positive cells and PCNA-positive cells were present at each age, indicating that the proliferation of male germ cells begins before the gonocytes migrate to the basement membrane of seminiferous tubules, which was consistent with the report on mice (Pinto et al., 2010). However, UCHL1-positive and Ki67-negative cells were present in 15-90-day-old dairy goat testes, corroborating that undifferentiated spermatogonia only partially have the ability to proliferate during pre-puberty, which was similar to the report on pigs (Zheng et al., 2022). Interestingly, SYCP3-positive cells were present in 90–240-day-old testes, indicating that male germ cells initiated meiosis (Pan et al., 2022). Besides, we also identified round spermatids that appeared in the 90-day-old samples, suggesting that dairy goat could reach puberty at 90 days after birth, which was consistent with our scRNA-seq data.

Single-cell RNA-seq can not only reveal the cell types involved in spermatogenesis, but also reveals the differentially expressed genes of each cluster to discover new marker genes (Vargo and Gilbert 2020). In this study, male germ cell and somatic cell marker genes were found by Loupe Cell Browser analysis, including SOHLH1, INHA, ACTA2, and SOX18. Wang et al. (2018) found that SOHLH1 is highly expressed in human SSCs and differentiating spermatogonia through scRNA-seq. Desimio et al. (2021) reported that SOHLH1 is generally considered expressed in C-KIT positive spermatogonia in mice. In this study, SOHLH1 was co-expressed with the germ cell marker VASA at the base of seminiferous tubules, suggesting that SOHLH1 can be used as a candidate marker gene for dairy goat spermatogonia, which is consistent with the reports for humans and mice (Wang et al., 2018; Desimio et al., 2021). An in-depth analysis of the expression profile of SOX transcription factors in mouse testes was conducted and determined that SOX18 is mainly expressed in spermatogonia (Roumaud et al., 2018). Moreover, SOX18 is mainly expressed in spermatocytes in sheep (Yang et al., 2021). We determined that SOX18 was mainly expressed in endothelial cells of goats, which indicated that the expression of SOX18 was different in mammalian testes. Additionally, INHA was highly expressed in Sertoli cell clusters, and INHA-positive cells in seminiferous tubules were VASA-negative, indicating that INHA may be a candidate marker gene for dairy goat Sertoli cells. Chaichanathong et al. (2018) performed immunochemical staining of adult Asian elephant testes and found that INHA is highly expressed in Sertoli cells, which is similar to our findings. ACTA2 has been reported as a marker gene for testicular somatic cells in mice and macaques (Kulibin and Malolina 2016; Sharma et al., 2019). We found that ACTA2 was highly expressed in Leydig cells and endothelial cells, suggesting that ACTA2 could be used as a candidate marker gene for testicular somatic cells in dairy goats.

Based on the cell trajectory analysis by Monocle, we analyzed the crucial genes associated with male germ cell development in dairy goats. The expression of many crucial genes is similar with spermatogenesis in humans and mice (Grive et al., 2019). DMRT1 is expressed in SSCs and Sertoli cells of mice, and participates in damage and repair of Sertoli and Leydig cells, thereby indirectly promoting the secretion of growth factors by testicular somatic cells. We found that DMRT1 is highly expressed in spermatogonia and Sertoli cells in dairy goats, which is consistent with previous studies (Zhang et al., 2016). Wei et al. (2021) reported that DMRT1 regulates the immune response by inhibiting the TLR4 signaling pathway in male goat germ cell lines, further confirming that DMRT1 plays an important role in spermatogenesis. Additionally, PIWIL1, TOP2A, NME8, PGK2, SYCP1, SYCP2, and ACR were highly expressed in dairy goat spermatocytes, which are similar to a report on human (Wang et al., 2018). When analyzing the G1/S and G2/M phase-specific genes, we found that the cell-cycle status of spermatogonia was very active in dairy goats. SSCs of mice are mostly in the G1 phase, partly in the S phase, and few in the G2/M phase (Wang et al., 2015). The cell cycle status of human SSCs is relatively inactive, and the G1/S and G2/M phase-specific genes are active in the differentiated spermatogonia (Wang et al., 2018). Additionally, GO and KEGG signaling pathways were enriched in male germ cells of dairy goats. We found the spermatogonia cluster was significantly enriched in RNA binding, protein complexes, and translation initiation factor activity-related pathways, and most differentially expressed genes were involved in the cell cycle. This was consistent with reports on sheep and humans, indicating that the differentiation process of mammalian spermatogonia has a certain similarity (Guo et al., 2018; Yang et al., 2021). These findings indicated that the G1/S and G2/M phase-specific genes play important roles in goat differentiated spermatogonia. Recently, Yang et al. (2021) conducted a functional enrichment analysis on the early spermatocytes of sheep, and significantly enriched in RNA splicing and ribonucleoprotein complex-related pathways, and most genes were involved in the cell cycle. These findings indicate that the differentiation process of mammalian spermatogonia is similar, and the dynamic changes of cell cycle-related gene expression in the spermatogonia and early spermatocyte are more active. Further confirmation of the mechanism and expression pattern is needed.

Sertoli cells can provide nutritional and morphological support for spermatogenesis by directly interacting with male germ cells (Yokonishi et al., 2020). However, little is known about the developmental patterns of Sertoli cells in dairy goats. Our scRNA-seq analysis provided a reference for understanding the development of Sertoli cells. We observed that AMH was only expressed in the clusters of GS45, suggesting AMH was only expressed in immature Sertoli cells of dairy goat testes. AMH and SOX9 have been reported as marker proteins of Sertoli cells in mice and humans (Grinspon and Rey, 2011; Banco et al., 2016). Interestingly, we found that AMH-positive and SOX9-positive cells were only observed in the 15 to 75-day-old samples, suggesting that AMH could be used as a marker protein of immature Sertoli cells in dairy goat, which is similar to previous reports (Zheng et al., 2022). Lara et al. (2020) analyzed the proliferation characteristics of testicular cells in a variety of animals and found that Sertoli cells usually mature before puberty and that mature Sertoli cells will eventually lose proliferation ability. In marmosets and Cebus monkeys, the proliferative activity of Sertoli cells increases rapidly during puberty, and the number of Sertoli cells remain homeostasis was found in post-puberty (Mäkelä et al., 2019). We identified that dairy goat reached puberty at 90 days after birth and Sertoli cells developed and matured without proliferation ability, which is consistent with previous reports (Zheng et al., 2022). Together, our study systematically investigated the elaborate developmental patterns of male germ cells and Sertoli cells in dairy goats.

Spermatogenesis involves the synergy of germ cells and somatic cells in the testes and the mutual transmission of a variety of signal molecules, and five niches (Sertoli, Leydig, myoid, endothelial, macrophage) have been reported in human and mice (Guo et al., 2018). Interestingly, Leydig cells have been reported that have a stronger paracrine effect on male germ cells than Sertoli cells (Qiao et al., 2022). Testosterone is mainly secreted by Leydig cells, lack of this hormone can cause hypogonadism and block spermatogenesis, which is essential for male development and maintenance of spermatogenesis (Gao et al., 2018). STAR, 3BHSD, and HSD3B7 are positively correlated with the secretion of testosterone (Eid et al., 2019; Zhou et al., 2020). In this study, they were highly expressed in the Leydig cell and Sertoli cell clusters of dairy goats. SRD5A1 plays a central role in the catabolism of testosterone, and the absence of SRD5A1 can lead to abnormal local or systemic androgen levels (Liu et al., 2020). In this study, testosterone response genes SRD5A1, SRD5A3, and SHBG were highly expressed in spermatogonia, early spermatocytes, Sertoli cells, and Leydig cells. It was suggested that testosterone as a paracrine factor diffused to Sertoli cells and germ cells to play a role in spermatogenesis of dairy goats, which is consistent with the report on the pathway of testosterone (Di Guardo et al., 2020). RA regulates spermatogenesis at many different stages, including driving the differentiation of spermatogonia and the beginning of meiosis, which is essential for spermatogenesis (Hogarth and Griswold 2013). ALDH1A1 and ALDH1A3, synthetase genes of RA (Raverdeau et al., 2012), are highly expressed in Sertoli cells and Leydig cells in this study. Additionally, the target gene STRA8 of RA is a key gene for the initiation of meiosis in spermatogonia, which was only highly expressed in spermatogonia of dairy goats in this study (Ferder et al., 2019). Deficiencies in STRA8 cause the lack of the ability to enter meiosis. It was previously reported that ALDH1A1 and ALDH1A3 are specifically expressed in human Leydig cells, whereas STRA8 was only observed during the transformation of spermatogonia into early spermatocytes, which is consistent with our results (Guo et al., 2018). Interestingly, we found that WNT2B, as a ligand of WNT, was highly expressed in the Leydig cells and early spermatocytes in dairy goats. However, WNT2B is mainly expressed in human testicular endothelial cells, and its response genes FZD3 and LBR3 are only expressed in early spermatocytes (Mamsen et al., 2017; Guo et al., 2018). Additionally, PDGFB has been reported to be expressed in human testicular endothelial cells, whereas its receptor genes PDGFRA and PDGFRB are expressed in Leydig cells, which is consistent with our findings in dairy goats (Guo et al., 2018). Our study revealed the potential key genes involved in the interaction between male germ cells and testicular somatic cells during spermatogenesis in dairy goats, providing a basis for detailed functional studies. Indeed, we have to acknowledge that our scRNA-seq data lacked biological repeats. Nevertheless, this study greatly adds to the body of knowledge on the developmental patterns of dairy goat male germ cells and Sertoli cells and lays the theoretical foundation for dairy goat breeding.

## Conclusions

In summary, our data represent the first comprehensive sampling and profiling of the developmental patterns of male germ cells and Sertoli cells in dairy goat from pre-puberty to post-puberty. The results revealed nine different germ cell clusters, four kinds of somatic cells, several marker genes, and the complex relationships of potential key genes in male germ cells and testicular somatic cells during spermatogenesis in dairy goats. The proliferative activity and development of male germ cells and Sertoli cells of dairy goats at different developmental stages has been clarified. Our findings not only offer fundamental transcriptome changes with regard to goat spermatogenesis, but also provide a rich resource regarding the developmental process of male germ cells and Sertoli cells.

## Data Availability

All datasets presented in this study are included in the article/Supplementary Material.

## Abbreviations

scRNA-seq, single cell RNA sequencing; SSCs, spermatogonial stem cells; HE, hematoxylin-eosin; PCA, principal component analysis; t-SNE, t-stochastic neighbor embedding; UMIs, unique molecular identifiers; GEM, gel beads-in-emulsion; GO, gene ontology; KEGG, kyoto encyclopedia of genes and genomes; EDTA, ethylenediamine tetraacetic acid; FITC-PNA, fluorescein isothiocyanate-peanut agglutinin; GS45, 45-day-old; GS90, 90-day-old; GS180, 180-day-old.
